# A modified targeted culturing approach provided a snapshot into interdependencies and resistome among core anaerobic bacteria of the healthy human gut

**DOI:** 10.1128/spectrum.03182-25

**Published:** 2025-12-31

**Authors:** ‌‌ Mahnoor, Riaz Ullah, Sonyia, Rawaiz Khan, Ali Bahadar, Ibrahim Elbatel, Imran Khan, Alaa Abdulaziz Alnahari, Adnan Haider, Syed Babar Jamal, Raees Khan

**Affiliations:** 1 Department of Biological Sciences, National University of Medical Sciences 445232, Rawalpindi, Pakistan; 2 Department of Restorative Dental Sciences, College of Dentistry, King Saud University 204573, Riyadh, Saudi Arabia; 3 King Salman Center for Disability Research 600567, Riyadh, Saudi Arabia; 4 Department of Chemical and Materials Engineering, King Abdulaziz University, Rabigh, Saudi Arabia; 5 Department of Mathematics and Statistics, College of Science, Imam Mohammad Ibn Saud Islamic University (IMSIU), Riyadh, Saudi Arabia; 6 Faculty of Medicine, Macau University of Science and Technology, Taipa, Macau, China; 7 Department of Biotechnology, Faculty of Chemical and Life Sciences, Abdul Wali Khan University Mardan 230180, Mardan, Pakistan; 8 Department of Biological Science, College of Science, University of Jeddah 441424, Jeddah, Saudi Arabia; Cleveland Clinic Lerner Research, Cleveland, Ohio, USA

**Keywords:** gut microbiome, core anaerobes, microbe-microbe interactions, culturomics, genomic insights, clinically relevant resistance

## Abstract

The core anaerobic component of the human microbiome plays a critical role in preserving the overall structure and function of the microbiome. However, how these core strains interact with each other and with other gut microbes is largely unknown. We employed a modified culturomics approach to selectively culture these core genera from the human gut and provided a snapshot into their ecological interactions, clinically relevant resistome, and whole genome sequence (WGS)-based insights into their observed phenotypes. This strategy successfully isolated representatives of the targeted 21 core genera, with some isolates reported for the first time. Cross-interactions and resistome-based profiling revealed important insights. Out of the 440 interaction assessments, a significant proportion exhibited either cooperative or competitive effects on other core genera. Interestingly, *Bacteroides thetaiotaomicron* displayed dual roles, inhibiting potential pathogens while enhancing the growth of beneficial probiotic strains. Moreover, resistome profiling revealed variable resistance and susceptibility toward various antibiotics. WGS analysis revealed the ability of the selected organism to produce metabolites consistent with the observed phenotype. Our unique culturomics approach successfully isolated key anaerobic genera and revealed their complex interdependencies and clinically relevant resistance. Furthermore, our approach lays the foundation for exploring larger-scale microbial community dynamics in terms of interdependencies and for assessing how clinically significant antibiotics impact the key microbiome, something that may be important when making therapy decisions. In summary, our research improves our knowledge of the interactions present among core gut microorganisms and hints at possible mechanisms responsible for sustaining gut microbial homeostasis and rehabilitation.

**IMPORTANCE:**

This study presents a unique strategy for the targeted isolation and characterization of key interaction dynamics as well as the resistome of core anaerobic bacteria in the healthy human gut. By utilizing this targeted single, optimized core anaerobe-specific growth medium approach, we were able to culture a major proportion of the core anaerobic gut species, where some of those were cultured for the first time. Through the combinatorial approach of cross-interaction, antimicrobial resistance profiling, and whole genome sequencing (WGS) resolved functional insights, we uncovered interesting insights into interdependencies and competition among these core strains as well as their ecological flexibilities. Moreover, we observed that some strains, such as *Bacteroides thetaiotaomicron* and *Bifidobacterium angulatum,* are capable of either inhibiting or promoting the growth of certain strains, depending on the pairing. Such findings go against the traditional binary view of microbes as being purely commensal or pathogenic and suggest that microbial behavior is highly context-dependent. Furthermore, WGS analysis provided useful insights into the potential genomic basis of the observed phenotype. Taken together, our findings provide valuable data and enhance our current understanding of how core anaerobes interact with each other, survive, and adapt within the gut environment. Moreover, our work also lays the critical basis for a more rational design of synthetic microbial communities as well as precision-based microbiome aimed at targeted gut health rehabilitation, both with accuracy and sensitivity.

## INTRODUCTION

The human gut is home to a diverse community of microorganisms, the microbiome that plays an important role in maintaining the host’s health. This host-associated microbiome impacts the health of the host by taking part in various metabolic processes, including nutrient metabolism, regulation of the host immune system, and providing direct or indirect protection against various pathogenic organisms (1). The bacterial component of the gut microbiome is dominated by anaerobic bacteria, which may constitute up to 99% of the community depending upon the location of the gastrointestinal tract (2). This anaerobic portion of the gut microbiome has been known to facilitate the host in various metabolic functions as well as retaining eubiosis (3–6).

Some members of the microbiome that play an important role in maintaining the overall structure and function of the microbial community are termed core microbiome components. This core microbiome component is gaining popularity in the scientific community as these core microbes are commonly found in healthy individuals (7–9). The core microbiome component is comparable to the queen in a bee colony, and the absence of these core microbes has been found to significantly delay the restoration of the gut microbiome or even lead to dysbiosis (10). For example, an analysis of large-scale microbiome data from four discovery cohorts across three continents—comprising over 500 profiles from 117 individuals—identified 21 bacterial species strongly associated with microbiome recovery following antibiotic therapy (10). However, the clinically relevant resistance and interactions among these core candidate genera are largely unexplored.

Understanding the co- and cross-interactions among core anaerobes and other gut microbes is crucial for identifying the factors that shape the overall community structure. The core microbiome has been explored in various review articles and metagenomics-based meta-analysis studies and declared as core based on their prevalence, stability, and functional importance (7, 11). Furthermore, targeting the core microbiome component has significant implications for synthetic microbiome transplants as it minimizes the bias and limitations associated with traditional microbiome transplantations, including pathogen transfer, transient results, and sustainable production (12, 13). Various anaerobic genera dominate the core microbiome component, including *Bacteroides*, *Clostridium*, *Faecalibacterium*, and *Prevotella*, and these are of particular interest due to their essential role in maintaining gut health by playing a role in many critical processes, such as fiber fermentation, short-chain fatty acid (SCFA) production, and immune modulation (14, 15). However, how these core genera are functionally dependent on each other, the nature of their co- and cross-interactions, and their clinically relevant antimicrobial resistance (AMR) remains poorly understood. This highlights the need for targeted approaches to explore their ecological and functional roles (16).

Recent advances in sequencing technologies have identified and defined the core taxa. However, sequencing technologies alone are unable to give valid insights into the overall functional capabilities of these core taxa, as they need to be validated *in vitro*. One of the major limitations so far in the characterization of the cross-interactions and clinically relevant resistome of these organisms is the culturing limitations. Since a major fraction of these core anaerobic bacteria is uncultured, this leads to a significant gap in our understanding of the human gut microbiome and the interactions within the microbial community (17). Microorganisms within a community interact with each other via various mechanisms, and these interactions are of immense importance for their survival within the community, as well as to modulate the overall microbial community dynamics (18). One of the traditional approaches for culturomics is the use of many different culture media types to selectively culture the targeted gut microbes. This non-economical and labor-intensive approach further necessitates the need for the development of unique representative media types, which can be utilized for the targeted culturing of the core bacteria. One ideal approach to design such media is mimicking the nutrient-rich conditions of the human gut. This specially designed media can then be utilized to investigate the interdependencies and cross-interactions among core microbes as well as among other anaerobes (19, 20).

Bacterial ecological interactions are broadly classified into several categories, with the most common being synergistic, antagonistic, and bifunctional. Synergistic interactions occur when co-cultured species mutually enhance each other’s growth and proliferate, often through cross-feeding of metabolites such as SCFAs, vitamins, or amino acids (21). Conversely, antagonistic interactions involve inhibitory effects that occur due to the secretion of bacteriocins or secondary metabolites, which suppress the proliferation of competitors and help define niche boundaries (22). Bidirectional interactions show context-dependent relationships where strains can have dual nature by promoting or inhibiting other’s growth due to relative abundances or nutrient-level dependencies (23).

Exploring the interdependencies in terms of co- and cross-interactions among the core members of the gut microbial community is of significant importance due to many reasons. Primarily, having an idea of how the core microbiome responds to clinically relevant antibiotics will further guide us regarding the impact of those antibiotics on the core taxa as well as on overall community structure (24). Additionally, such profiling will help clinicians in informed decision-making prior to prescribing antibiotics and selected alternatives with potential minimal impact on the gut microbiome. Moreover, the knowledge of co- and cross-interactions among community members will help us gauge which types of interactions govern the community dynamics and are responsible for maintaining the overall structure and function of the community (25).

Here, we have developed a unique culturomics strategy by utilizing a core anaerobe-specific growth medium (CASGM). Using CASGM, we targeted the core anaerobic fraction of the healthy human gut microbiome. This approach has the advantage as it bypasses the need for using diverse culture media types. Furthermore, using CASGM, we gave a snapshot into the cross-interactions among these core anaerobes and also determined their interactions with other members of the community. Additionally, using this approach, we explored the clinically relevant resistome of these organisms. In addition, we have correlated the observed phenotype with potentially responsible genetic elements by using a combinatorial approach of CASGM and whole-genome sequencing (WGS), which provided valuable insights into observed interdependencies (26, 27). In summary, our CASGM-based modified culturomics approach can be utilized to selectively culture core anaerobes and provide a snapshot of their resistome and interdependencies, thus shedding light on this largely unexplored research area, which is in its embryonic stage and needs to be explored.

## MATERIALS AND METHODS

### Screening and selection of healthy human donors

Healthy human donors (age range: 18–40 years old) were selected on the basis of standardized questionnaires, and their fecal samples were taken with informed consent. Some of the main inclusion criteria included no use of antibiotics and probiotics by the donor in the last 6 months, with no current or previous history of any gastrointestinal inflammatory disorders. Donors meeting all the criteria were included in the study, and their fecal materials were collected in sterile containers, immediately aliquoted, and processed for bacterial isolation. These aliquots were also stored in 40% glycerol at −80°C for backup and future processing.

### Isolation of core anaerobic bacteria

In this study, two biological replicates were processed at different time points for isolation and culturing. We designed a specialized phosphate buffer solution (PBS) by adding ascorbic acid (0.01 g/L), which is a strong antioxidant. We named this buffer as the “anaerobe viability-supportive PBS (AVSPBS)” and will be referred to as AVSPBS hereafter. The collected stool samples were suspended in AVSPBS and were homogenized to make a uniform suspension. In summary, we suspended a total of 1 g of fresh fecal material in 9 mL of AVSPBS in a sterile Falcon tube. This mixture was then homogenized using a vortex to make a uniform suspension and was serially diluted. Specifically, successive 10-fold dilutions, ranging from 1 × 10⁻¹ to 1 × 10⁻¹⁰, were prepared by transferring 100 µL of the suspension mixture into 900 µL of fresh AVSPBS. Each dilution was carefully mixed to ensure uniform bacterial distribution. We isolated anaerobic bacterial species by handling the samples in biosafety level 2, which is the recommended biosafety level for handling human gut microbiota. Contaminant measures, such as autoclaving media and spreaders at 121°C for 15 min, filtering antifungal and antibiotic solutions, sterilizing the biosafety cabinet environment with UV light, pouring media inside the biosafety cabinet, wearing lab coats and gloves, and sterilizing hands with 70°C ethanol, were taken. These measures were adapted during the isolation and purification processes to avoid contamination. Furthermore, to minimize or exclude potential cross-contamination of stool samples, each individual’s fecal samples were processed in a separate biosafety cabinet along with other containment measures as mentioned above.

### Preparation of CASGM

To target the culturing of core anaerobes from healthy donors’ fecal samples, we designed a modified complex growth medium, termed as CASGM. The CASGM was formulated by adding ascorbic acid (1 g/L) and L-cysteine (0.2 g/L) as additional antioxidants. In summary, the culture media were prepared by adding all the ingredients (Table 1) in a sterile flask. L-cysteine (0.2 g) was added to 50 mL of 1 N HCl and mixed thoroughly. Then, the remaining components and 950 mL of distilled water were added. The pH was adjusted to 7.6–7.8, and the media were sterilized by autoclaving at 121°C for 15 min. Upon cooling (at ≈50°C), 5% horse blood (50.0 mL) and 1% ascorbic acid (10.0 mL) were added aseptically (Table 1).

**TABLE 1:** Composition of CASGM

Sr. no.	Ingredients	Amount
1	Lab-Lemco Powder (Oxoid)	2.4 g
2	Protease Peptone No. 3 (BD-Difco)	10.0 g
3	Yeast Extract (BD-Difco)	5.0 g
4	Sodium phosphate dibasic (Na_2_HPO_4_)	4.0 g
5	Glucose (dextrose)	1.5 g
6	Soluble starch (succinic anhydride)	0.5 g
7	L-cysteine	0.2 g
8	1 N HCL	50.0 mL
9	Agar	15.0 g
10	L-cysteine·HCl·H_2_O	0.5 g
11	Horse blood	50.0 mL
12	Distilled water	Add to reach a final volume of 1-L
13	Ascorbic acid	10.0 mL

### Screening and shortlisting of core anaerobic bacteria

Anaerobic serial dilutions in AVSPBS were further proceeded using the spread plate method by evenly spreading 100 µL from each dilution onto CASGM culture media plate, and the plates were kept in an anaerobic jar with Oxoid AnaeroGen 2.5L packs (Thermo Scientific) and Oxoid Resazurin Anaerobic indicator (Thermo Scientific) to ensure anaerobic conditions. We used Resazurin redox dye strip because of its low toxicity toward microorganisms and its high efficiency even at low concentrations (1–2 µg mL^−1^) (28). An anaerobic jar system was used to isolate core anaerobic bacteria, with suitable positive and negative control bacteria to ensure that the jar was providing the required environment for culturing anaerobic bacteria. The jar was then tightly closed to avoid oxygen exposure during the incubation period. The jar was incubated at 37°C for 48 h, but additional incubation of 24 h was continued to support the growth of slow-growing core anaerobes. Pure isolates were shortlisted on the basis of colony morphology, and a single unique colony was processed further to avoid duplication.

### Optimizing conditions for cryopreservation of the core anaerobes

Obligate anaerobes are highly sensitive to any available oxygen; therefore, to ensure their viability during cryopreservation, we used a modified glycerol solution. Cryoprotectants and their sterilization conditions include 40% glycerol (autoclaved at 121°C for 20 min), amended with 0.2-micron syringe-filtered ascorbic acid (1 g/L) and glutathione (0.1 g/L). The purified colonies were preserved in this modified 40% glycerol stock and stored at −80°C. The glycerol stocks were checked regularly every month to ensure the viability of anaerobes.

### DNA extraction, 16S rRNA gene amplification, and identification of core anaerobes using Sanger sequencing

DNA extraction was performed using two different methods: the boiling method and a commercial DNA extraction kit (Solarbio). The 16s rRNA gene (V3–V4) region was amplified by using universal primers 341-F (5′ CCTACGGGNGGCWGCAG 3′) and 805-R (5′ GACTACHVGGGTATCTAATCC 3′). The PCR conditions included an initial denaturation at 95°C for 15 min, followed by 25 amplification cycles of denaturation (1 min at 95°C), annealing (25 s at 47.8°C), and extension (30 s at 72°C), with a final extension at 72°C for 5 min. The PCR products were analyzed on a 1.5% agarose gel in 1× TBE buffer (45 mM Tris-borate and 1 mM EDTA) at 120 V for 90 min using the BIORAD ChemiDoc XRS system. The amplified 16S rRNA gene products were sequenced using Sanger sequencing technology (Macrogen, South Korea). The sequence data were analyzed using BioEdit, EzBioCloud, and NCBI databases for the identification of core anaerobic bacterial strains. Initially, several hundred isolates were identified via 16S ribosomal RNA V3–V4 region. Later on, selected isolates were processed for WGS, and along with species-level identification validation, genomes were annotated for metabolites implicated in antagonistic and synergistic interactions.

### Clinically relevant AMR profiling of core anaerobes

To evaluate the impact of clinically relevant antibiotics on representative core anaerobic genera, we conducted AMR profiling. The rationale for selecting these eight antibiotics was their clinical relevance and their representation of eight different antibiotic categories, which are broadly used against a variety of infections. Using the drop method, a set concentration of 15 µg/mL for eight classes of antibiotics—oxytetracycline, meropenem, ampicillin, chloramphenicol, ceftriaxone, levofloxacin, azithromycin, and tobramycin—was prepared in CASGM. Core anaerobic strains were suspended in AVSPBS to achieve an optical density (OD) ranging from 0.1 to 0.12 at 600 nm. From this suspension, 1.5 µL of each strain was carefully spotted onto plates with and without antibiotic supplementation. Incubation was carried out at 37°C for 48 h, with regular inspection of the plates to monitor zones of inhibition. Control plates, devoid of antibiotics, served as baselines for comparative analysis. Each AMR assay was conducted in three technical replicates, with positive and negative controls.

### Assessment of cross-interactions among anaerobes

To investigate interspecies growth interactions among core anaerobic strains, we utilized the agar overlay method. Each strain was prepared in a homogeneous suspension using AVSPBS, with an OD standardized between 0.1 and 0.12 at 600 nm. A 100 µL aliquot of each suspension was evenly spread onto CASGM using sterile cotton swabs to create a uniform bacterial lawn. Wells were then formed in the same CASGM plates, and 100 µL of other core anaerobic strains was introduced into these wells. Control plates, containing only the wells with bacterial strains and no overlay, were prepared similarly. All plates were carefully placed in an anaerobic jar to prevent any splashing or spills, followed by incubation at 37°C for 48 h. Each strain was processed in three technical replicates. Upon completion of incubation, results were evaluated by comparing test plates with controls: an enhancement in bacterial growth around the wells indicated a synergistic interaction, while the presence of clear inhibition zones around the wells signified antagonistic activity.

### Analysis of *Bifidobacterium angulatum* WGS reads

Purified isolate of the selected anaerobe, *B. angulatum,* having a unique interaction profile, was sequenced in collaboration with GetGenome (UK) using third-party sequencing services. A web-based pipeline of the DOE Systems Biology Knowledgebase (Kbase) was used for analysis. Raw reads were processed for quality control assessments using the FastQC tool. PRINSeq, Trimmomatic, and Bloom Filter Corrector were applied for filtering low-complexity reads, trimming adapters, and removing sequencing artifacts, respectively. High-quality contiguous sequences were processed for *de novo* assembly using SPAdes with multi-kmer strategy. The genome assembly was evaluated for completeness and contamination using QUAST and CheckM, respectively. Complete (>95%) genome with <5% contamination was taxonomically classified through GTDB and annotated via RAST. An annotated assembly was processed for metabolic modeling using the Build Metabolic Model tool and KEGG pathways to elucidate potential genetic determinants of microbe-microbe interactions.

## RESULTS

### Targeted culturing of core anaerobes

To culture the targeted core anaerobes, we initially isolated 49 anaerobic strains from healthy fecal donor samples using our CASGM-based targeted culturing approach. To optimize resources and reduce sequencing costs, we performed preliminary differentiation based on colony morphology, allowing us to identify and retain only unique isolates. This screening resulted in 24 distinct anaerobic isolates, which were subsequently sent for identification via Sanger sequencing and further analysis.

### Successful culturing of core anaerobes with CASGM-based approach as confirmed by 16S rRNA-based identification

**Fig 1:**
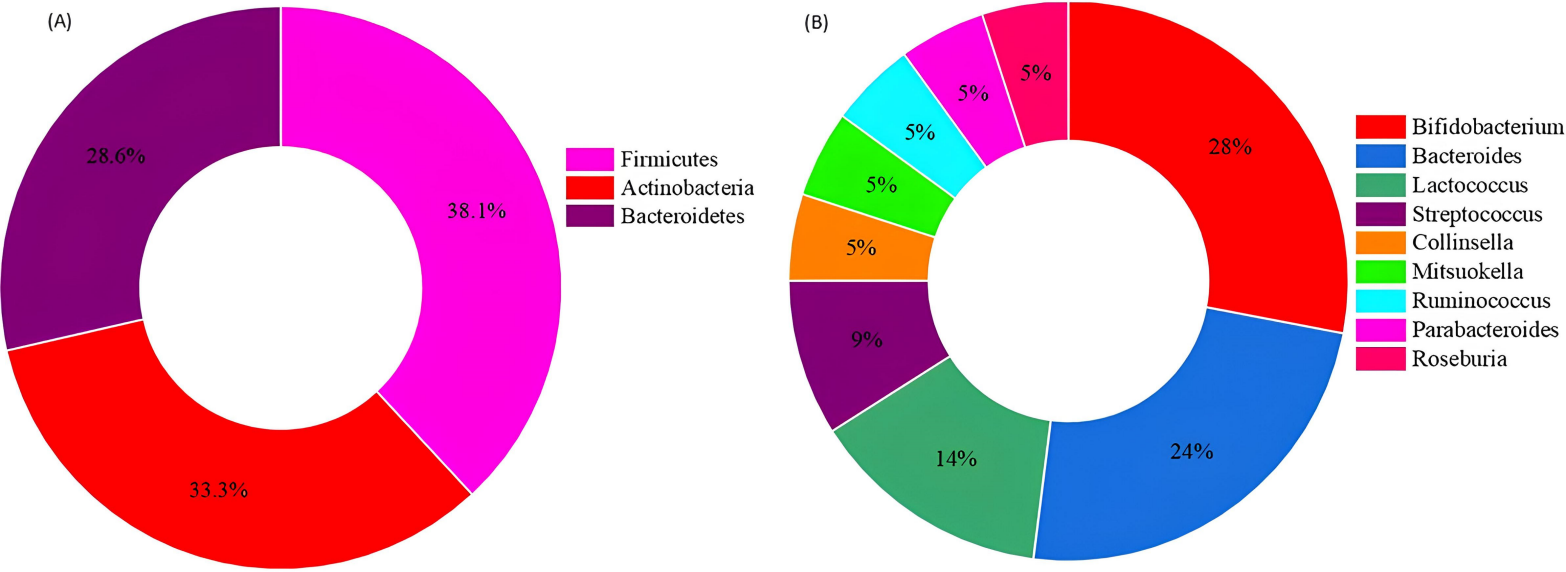
Percentage distribution of identified anaerobic isolates according to (**A**) phylum, (**B**) genus.

Using the CASGM-based culturing approach, we successfully isolated differentially abundant species, including 21 anaerobes, of which 11 were targeted core anaerobic species (Table 2), representing 52.4% of the species we aimed to culture. The complete list of isolates is provided in Table 2. These isolates represented a range of phyla, including Firmicutes (38.09%), Actinobacteria (33.3%), and Bacteroidetes (28.57%) (Fig. 1A). The isolates were further classified into nine distinct genera, with *Bifidobacterium* constituting the highest abundance at 28.57%, followed by *Bacteroides* and *Lactococcus*, each at 23.8%. Other identified genera included *Streptococcus* (9.52%) and *Collinsella*, *Mitsuokella*, *Ruminococcus*, *Parabacteroides*, and *Roseburia*, each contributing 4.76%.

**TABLE 2:** Core anaerobic bacteria cultured via CASGM^*a*^

S. no.	Core anaerobe (10)	Cultured via CASGM in our study
1	***B. uniformis***	**Yes**
2	*Alistipes putredinis*	No
3	*Alistipes shahii*	No
4	***B. thetaiotaomicron***	**Yes**
5	*Parabacteroides distasonis*	No
6	*Coprococcus catus*	Yes
7	***B. adolescentis***	**Yes**
8	*Ruminococcus bromii*	No
9	*Subdoligranulum variable*	No
10	*Bacteroides stercoris*	No
11	*Bacteroides eggerthii*	No
12	***B. coprocola***	**Yes**
13	***B. bifidum***	**Yes**
14	***R. inulinivorans***	**Yes**
15	***B. caccae***	**Yes**
16	*Faecalibacterium prausnitzii*	No
17	***R. torques***	**Yes**
18	***B. longum***	**Yes**
19	***B. intestinalis***	**Yes**
20	*Desulfovibrio piger*	No
21	***P. johnsonii***	**Yes**

^*a*^ Yes, the corresponding core anaerobe was successfully cultured via our CASGM approach. No, the corresponding core anaerobe was not cultured via our CASGM approach. The strain names highlighted in bold also represent the successful culturing of these core anaerobes via our CASGM.

The majority of these isolates, 66.67%, were obligate anaerobes, followed by 19.04% facultative anaerobes, 9.52% microaerophilic anaerobes, and 4.76% microaerotolerant anaerobes. Specifically, the obligate anaerobes included species such as *Collinsella aerofaciens*, *Bifidobacterium pseudocatenulatum*, *B. angulatum*, *Bifidobacterium adolescentis*, *Mitsuokella multacida*, *Bifidobacterium bifidum*, *B. thetaiotaomicron*, *Ruminococcus torques*, *Bacteroides caccae*, *Bacteroides coprocola*, *Bacteroides intestinalis*, *Bacteroides uniformis*, *Parabacteroides johnsonii*, and *Roseburia inulinivorans*. Facultative anaerobes included *Lactococcus garvieae*, *Lactococcus fujiensis*, *Lactococcus formosensis*, and *Streptococcus equinus*, while microaerophilic anaerobes included *Streptococcus anginosus* subsp*. anginosus* and *Bifidobacterium longum* subsp*. infantis*. Finally, *Bifidobacterium longum* subsp*. longum* was identified as a microaerotolerant anaerobe.

This selective culturing successfully cultured core anaerobic genera, underscoring the method’s capacity to support diverse yet targeted bacterial communities and to promote insights into the composition and functional potential of anaerobic microbiomes.

### Expansion of the cultured gut anaerobes via CASGM approach

Our targeted culturing approach successfully isolated 11 core anaerobic species along with other important anaerobes from the healthy human gut microbiome. Interestingly, 19.04% of these anaerobes—namely, *L. fujiensis*, *L. formosensis*, *S. anginosus* subsp. *anginosus*, and *L. garvieae*—have not been previously isolated from the human gut. This study marks the first successful isolation of these strains from healthy individuals using the CASGM approach (Table 3).

**TABLE 3:** Previous isolation niches of the anaerobes isolated from the human gut via CASGM approach

Species name	Isolated for the first time in our study from human gut (yes/no)	Previous isolation niches	References
*C. aerofaciens*	No	Oral microbiome, human fecal sample	(29)
*B. pseudocatenulatum*	No	Human fecal samples	(30)
*L. garvieae*	Yes	Affected rainbow trout fish, dairy products, meat products, vegetables, cereals, silage, raw milk	(31, 32)
*B. angulatum*	No	Breast-fed infants’ fecal sample/human feces	(33)
*B. adolescentis*	No	Infant fecal sample, human milk, bovine rumen	(34)
*B. longum* subsp*. longum*	No	25-year-old healthy Korean female, milk-based drink, yogurt, infant formula, gut of a healthy breast-fed infant in 1969	(35)
*M. multacida*	No	Endocarditis patient fecal sample, piglet feces, human feces/swine feces	(36)
*L. fujiensis*	Yes	Chinese cabbage	(37)
*L. formosensis*	Yes	Yan-tsai-shin (fermented broccoli stem), fermented soybean meal	(38)
*S. anginosus* subsp*. anginosus*	No	Human body	(39)
*B. longum* subsp*. infantis*	No	Healthy fecal sample/infant fecal sample	(40)
*B. bifidum*	No	Fermented food, i.e., yogurt, human fecal sample	(41)
*B. thetaiotaomicron*	No	Healthy adult fecal sample, cow, pig, goat, mouse	(42)
*R. torques*	No	Initially from human gut, bovine rumen, cecum of the feral chicken	(43)
*B. caccae*	No	Human fecal sample, blood sample	(44)
*B. coprocola*	No	Human fecal sample	(45)
*B. intestinalis*	No	32-year-old Japanese female fecal sample	(46)
*B. uniformis*	No	Human wound, healthy human colon, stool of healthy breast-fed infants	(47, 48)
*P. johnsonii*	No	Japanese human fecal sample	(49)
*R. inulinivorans*	No	Human fecal sample in an M2 medium-based culture system	(50)
*S. equinus*	No	Fresh horse dung, cattle, and human feces	(51)

For instance, *L. fujiensis* was initially isolated from Chinese cabbage, while *L. formosensis* was first identified in Yan-tsai-shin (fermented broccoli stem) and subsequently in fermented soybean meal (38). *L. garvieae* was originally isolated from diseased rainbow trout and has since been found in dairy products, meat products, and vegetables. In contrast, there are limited isolation data available for *S. anginosus* subsp. *anginosus*. To date, no prior studies have confirmed the isolation of these strains from the healthy human gut (Table 3).

### Functional classification based on their potential roles indicated that the majority of the isolated core anaerobes possess probiotic potential with some potential pathogens

To classify the isolated core anaerobes based on their potential roles within the gut microbiome, we conducted a comprehensive review of previously published, well-established studies to categorize each isolate as either a potential probiotic or a potential pathogen. This classification took into account each isolate’s physiological functions and any associations with disease etiology.

Our analysis revealed that 66.6% of the isolated anaerobes potentially exhibit probiotic potential. Among the probiotic candidates, strains such as *C. aerofaciens*, *B. pseudocatenulatum*, *B. angulatum*, *B. adolescentis*, *L. formosensis*, *B. longum* subsp*. infantis*, *B. bifidum*, *R. inulinivorans*, *B. coprocola*, *B. intestinalis*, *B. uniformis,* and *M. multacida* demonstrate significant probiotic attributes. *P. johnsonii* also exhibits probiotic potential; however, it was concurrently classified as a potential rare pathogen due to its increased prevalence in patients with alopecia.

Others were classified as potential pathogenic strains, i.e., *S. anginosus* subsp*. anginosus* and *L. garvieae*, the latter being a known zoonotic pathogen. Moreover, *R. torques*, a species known for its dual nature, potentially acts as both a probiotic and exhibits pathogenic effects. However, current data are insufficient to conclusively support its pathogenicity. Due to inadequate studies, *L. fujiensis* and *S. equinus* were considered non-pathogenic strains.

### Snapshot of clinically relevant AMR indicates high susceptibility to clinically relevant antibiotics

**Fig 2:**
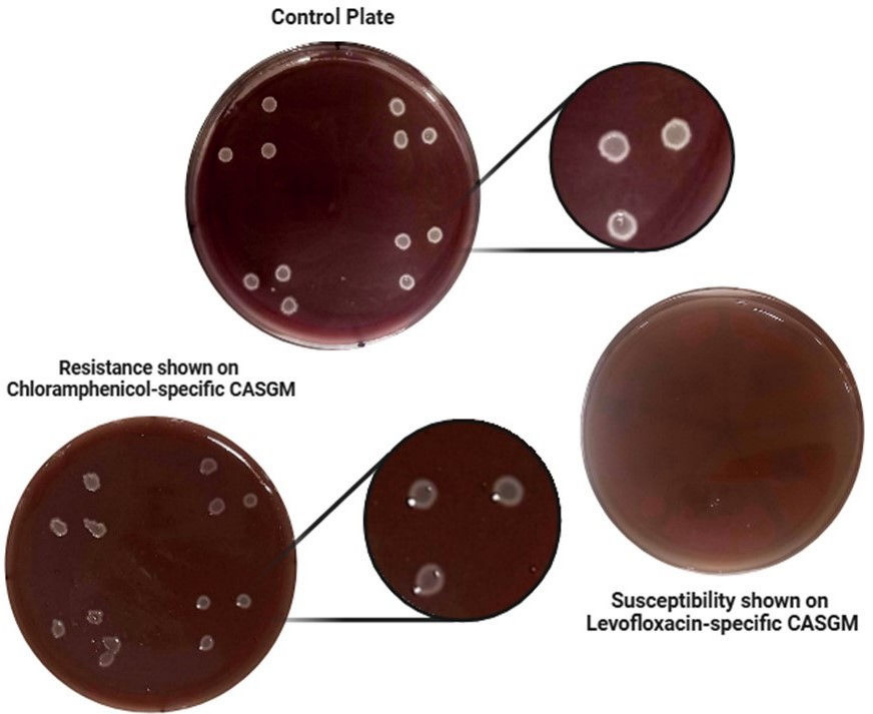
Representative picture of AMR profiling of core anaerobes against different tested antibiotics.

Antimicrobial susceptibility was evaluated by comparing bacterial growth on antibiotic-supplemented plates to that on control plates (Fig. 2). Strains whose growth was completely inhibited are termed susceptible, whereas those showing slight growth were deemed moderately resistant, and the strains whose growth was not inhibited are referred to as resistant.

**Fig 3:**
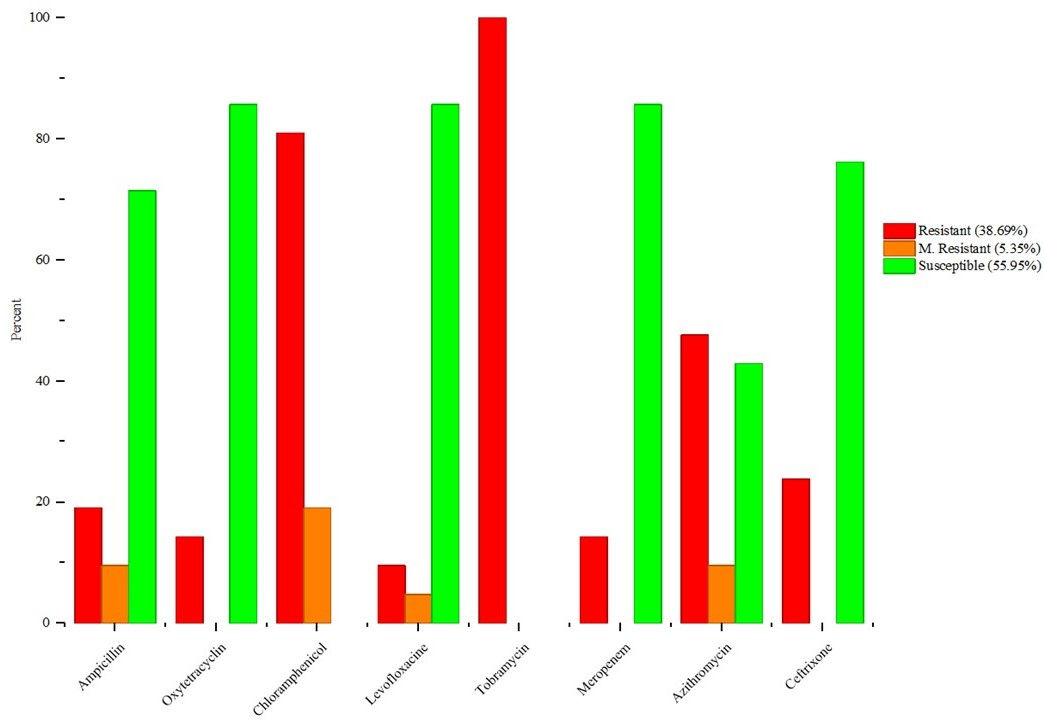
The combined clustered column chart illustrates the susceptibility profiling of isolated bacterial strains categorized as resistant, MR, and susceptible to antibiotics. The red color presents resistance, and the orange color shows MR, while the green color denotes sensitivity.

AMR profiling revealed that 55.95% were susceptible to the eight tested antibiotics. This indicates that over half of the core anaerobic bacterial population can be targeted by standard clinically relevant antimicrobial treatments and may cause dysbiosis. Meanwhile, 38.69% of the isolates demonstrated resistance, suggesting the presence of adaptive mechanisms that facilitate survival despite antibiotic exposure. Additionally, 5.35% of the anaerobic strains exhibited moderate resistance (MR), which may become susceptible at high antibiotic dosages (Fig. 3).

Detailed analysis revealed that 100% of the 21 strains were resistant to tobramycin, followed by 80.95% resistance to chloramphenicol. A notable proportion of isolates also exhibited resistance to azithromycin (47.61%), a member of the macrolide class. In contrast, the highest susceptibility rates of core anaerobes were shown against antibiotics such as meropenem, levofloxacin, and oxytetracycline (Fig. 3).

**Fig 4:**
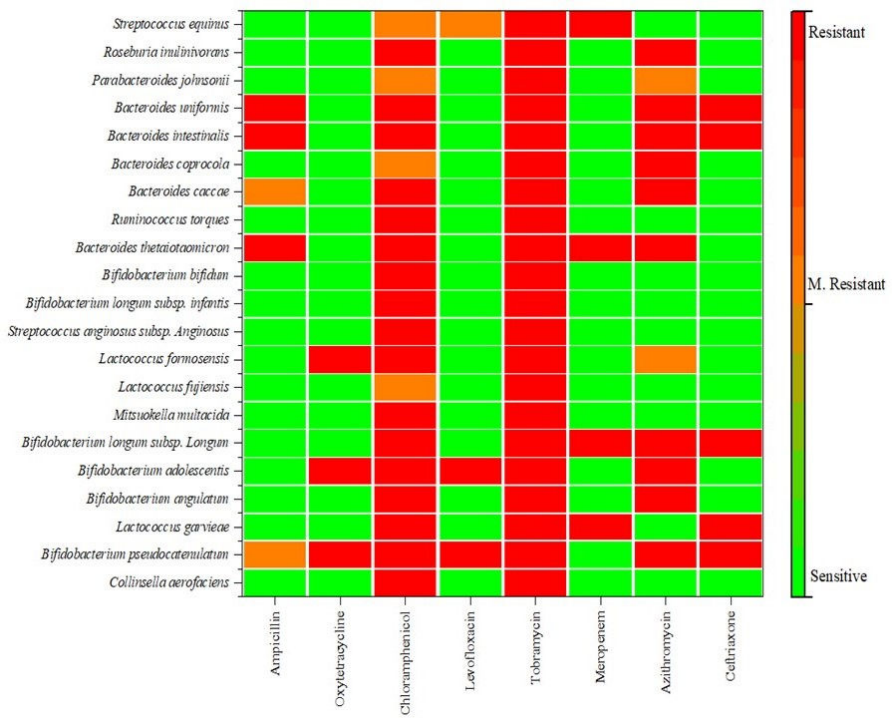
Heatmap of antibiotic susceptibility, resistance, and MR of the anaerobic isolates, including core anaerobes against eight different classes of antibiotics. Red color shows resistance, orange color shows MR, and green color shows sensitivity.

Chloramphenicol (19.09%) depicted the highest ratio of MR, followed by azithromycin and ampicillin (9.53% each) (Fig. 3). *B. pseudocatenulatum* and *B. caccae* were MR to ampicillin, *L. formosensis* and *P. johnsonii* are MR to azithromycin, whereas *L. formosensis, B. coprocola, P. johnsonii,* and *S. equinus* exhibited MR to chloramphenicol (Fig. 4).

Some of the organisms showing resistance to at least one agent in three or more antimicrobial categories were considered multidrug resistant. These organisms include *B. pseudocatenulatum*, *L. garvieae*, *B. angulatum*, *B. adolescentis*, *B. longum* subsp*. longum*, *B. thetaiotaomicron, L. formosensis, B. caccae, B. coprocola, B. intestinalis, B. uniformis, P. johnsonii, R. inulinivorans, and S. equinus* (Fig. 4).

### Snapshot of cross-interactions among core anaerobes revealed synergistic, antagonistic, and bifunctional impacts on probiotic and pathogenic strains

To have an idea of the synergistic and antagonistic activities of all isolated anaerobes, cross-interaction studies were performed. A total of 440 microbial interactions were studied, where 31 synergistic and 35 antagonistic responses were noticed. Most importantly, 33.33% of anaerobes showed both synergistic and antagonistic behaviors. This dual role indicates the complexity of microbial interactions, where certain strains can promote the growth of some taxa while simultaneously inhibiting others. Overall, 80.93% of the isolated anaerobes revealed either synergistic or antagonistic interactions.

**Fig 5:**
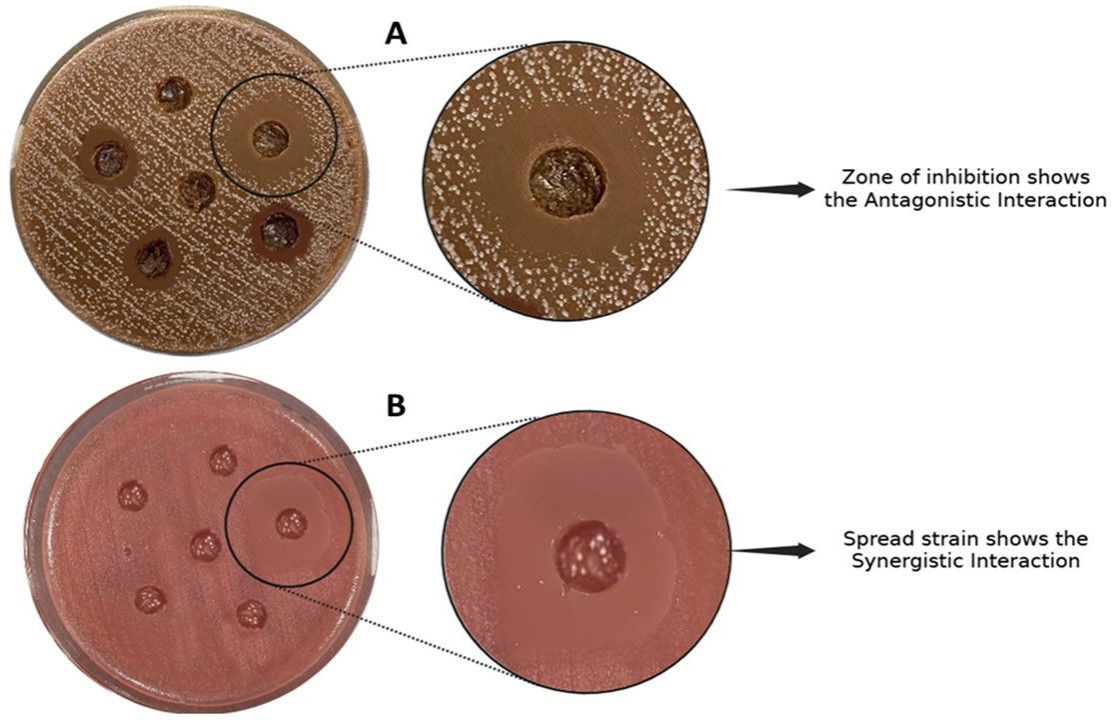
Representative pictures of antagonistic and synergistic interactions among gut anaerobes. The figure demonstrates antagonistic and synergistic interactions between isolated anaerobes. (**A**) Antagonism is illustrated by the presence of a zone of inhibition, which indicates the suppression of growth in the surrounding areas. (**B**) Synergism is evidenced by enhanced growth around the wells.

**Fig 6:**
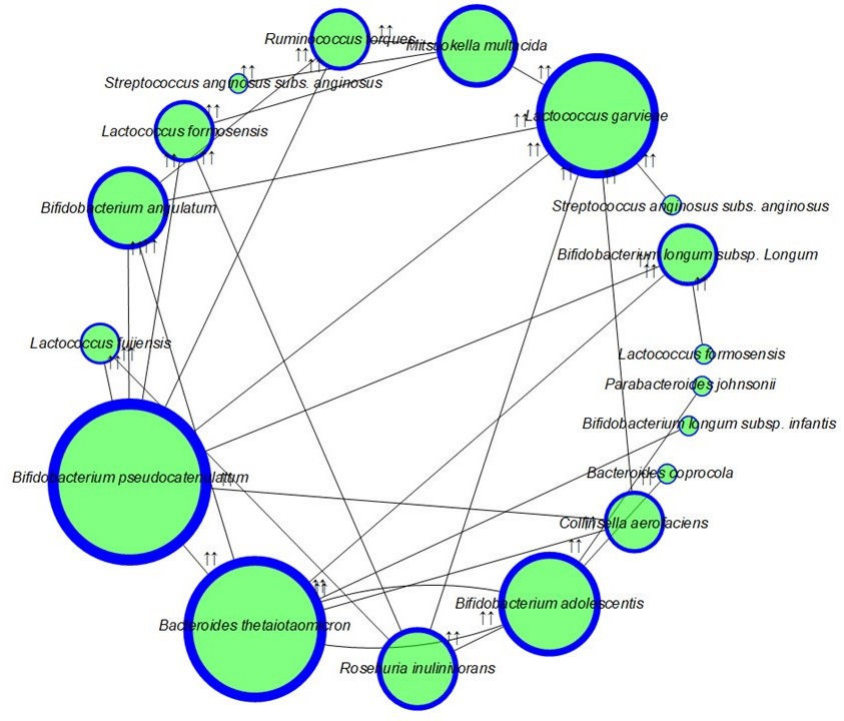
Interaction network showing species growth enhancement by helper species among anaerobic isolates. Size varies with the number of interactions, i.e., larger size demonstrates more interactions, while smaller size shows one or two interactions. The largest size source node shows interactions with eight other species, and the smallest size source node attributes a single interaction. Double upward arrows near the target node present the species whose growth was enhanced, while the emerging edge link from the source node denotes the corresponding helper species.

**Fig 7:**
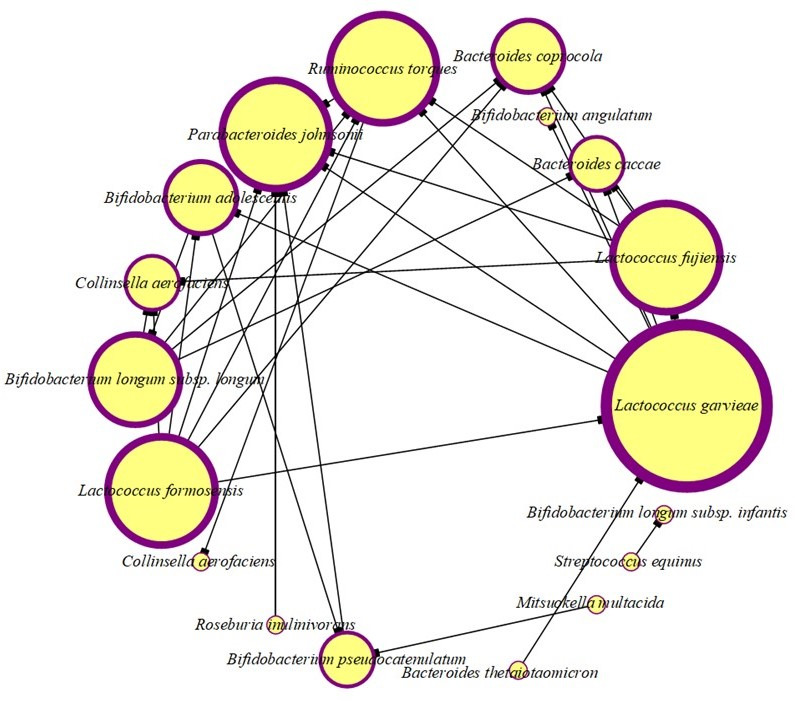
Interaction network showing species growth inhibition by antagonistic species among anaerobic isolates. The size of the circle represents the number of interactions: the largest size shows the maximum, and the smallest size illustrates the minimum number of interactions among the species. The inhibition symbol at the end of the link indicates the species whose growth was inhibited, while the emerging link without the inhibition symbol denotes the antagonistic species responsible for inhibition.

Isolates showing synergistic potential (Fig. 5B) were termed as helper strains, which include *L. formosensis, S. anginosus* subsp*. anginosus, M. multacida, B. longum* subsp*. longum, B. pseudocatenulatum, C. aerofaciens, B. adolescentis, B. angulatum, R. inulinivorans,* and *B. thetaiotaomicron. B. pseudocatenulatum* showed the highest number of synergistic interactions impacting the growth of seven other anaerobic strains. Other major helper strains that enhanced the growth of ≥3 other strains include *M. multacida, R. inulinivorans, B. thetaiotaomicron,* and *B. adolescentis*. Out of these 12 helper strains, 7 strains showed “both” synergistic and antagonistic interactions. These helper strains enhanced the growth of important gut bacteria, such as *L. formosensis,* which enhanced the growth of the multifunctional probiotic strain *B. longum* subsp*. longum. B. pseudocatenulatum* promoted the growth of several probiotics, while inhibiting *P. johnsonii*. Similarly, *B. pseudocatenulatum* enhanced the growth of *L. fujiensis, B. longum* subsp*. longum, and R. torques* (Fig. 6 and 7). Interestingly, the potential pathogenic bacteria *B. thetaiotaomicron* showed a synergistic effect on the growth of four probiotic bacteria, including *B. longum* subsp*. longum, B. adolescentis, B. angulatum,* and *C. aerofaciens* (Fig. 6).

### Core anaerobic isolates facilitated the growth of potential pathogenic taxa through cross-feeding interactions

Interestingly, several core anaerobes traditionally regarded as probiotic or potentially probiotic, such as *Bifidobacterium* strains*, M. multacida, R. inulinivorans,* and *C. aerofaciens*, were found to significantly enhance the growth of pathogenic, potentially pathogenic, and zoonotic species (Fig. 6 and Table 4). For example, all of the mentioned strains supported the growth of *L. garvieae*. Additionally, various *B. adolescentis, B. pseudocatenulatum*, and *C. aerofaciens* enhanced the growth of the potential zoonotic pathogen *B. thetaiotaomicron* (Fig. 6 and Table 4). Moreover, *M. multacida* also enhanced the growth of the pathogenic bacteria *S. anginosus* subsp*. anginosus*. This observation highlights an important yet underappreciated duality, where beneficial strains, through metabolic cross-feeding or environmental modulation, may inadvertently promote the growth of undesirable or harmful microbes. Such findings challenge the conventional view of probiotics as unilaterally beneficial and underscore the necessity of evaluating microbial interactions at the strain level within defined community contexts. These results carry critical implications for the rational design of microbiome-targeted therapies and raise caution against generalized assumptions in probiotic applications.

**TABLE 4:** Each species and its potential as reported in previous scientific literature

Species	Potential	References
*B. coprocola*	Potential probiotic	(52)
*B. caccae*	Potential pathogen	(53)
*B. adolescentis*	Probiotic	(54)
*B. angulatum*	Probiotic	(55)
*B. longum* subsp*. infantis*	Probiotic	(56)
*B. longum* subsp*. longum*	Probiotic	(35)
*B. pseudocatenulatum*	Probiotic	(57)
*C. aerofaciens*	Probiotic	(29)
*L. fujiensis*	Non-pathogen	(37)
*L. garvieae*	Zoonotic pathogen	(58)
*P. johnsonii*	Pathogen	(59)
*R. torques*	Not known	

### Synergistic interactions revealed interesting interdependencies when the growth media playing field was changed

A notable bidirectional growth-promoting interaction was observed between *B. thetaiotaomicron* and *B. adolescentis*. When the playing field was altered—by pre-spreading one strain across the agar surface and inoculating the other into a central well—each strain enhanced the growth of the other in both configurations. *B. thetaiotaomicron* facilitated the growth of *B. adolescentis* when used as the background strain, and reciprocally, *B. adolescentis* promoted *B. thetaiotaomicron* under reversed conditions (Fig. 6). This mutualistic interaction suggests a potential cross-feeding or environmental modulation mechanism that is robust across positional contexts, underscoring their ecological interdependence within the gut microbiota.

### Antagonistic interactions revealed pathogen-pathogen, probiotic-pathogen, and pathogen-probiotic antagonism potential, along with other interdependencies

Isolates showing antagonism against ≥3 strains were termed as major antagonists, and this group was dominated by *Lactococcus* species. Of these, *L. garvieae, L. formosensis,* and *L. fujiensis* showed the highest number of antagonistic interactions by inhibiting the growth of seven, six, and five strains, respectively (Fig. 7 and Table 4). Some of the potential pathogenic isolates inhibited the growth of other potential pathogens, such as potentially pathogenic *L. garvieae,* which inhibited the growth of *B. caccae* and *P. johnsonii. L. garvieae* also inhibited the growth of various potentially probiotic genera, including *Bifidobacterium, Lactococcus,* and *Bacteroides*. Other isolated strains showing inhibition of potential pathogens include *L. formosensis*, *L. fujiensis, B. longum* subsp*. longum, B. pseudocatenulatum, R. torques,* and *R. inulinivorans*. Some probiotic potential isolates antagonized the growth of other potentially probiotic strains (Fig. 7 and Table 4). Strains against which high antagonism was observed include *B. caccae, B. coprocola, R. torques, P. johnsonii,* and *C. aerofaciens*.

### Genomic insights into *B. angulatum* focusing on microbe-microbe interactions

**Fig 8:**
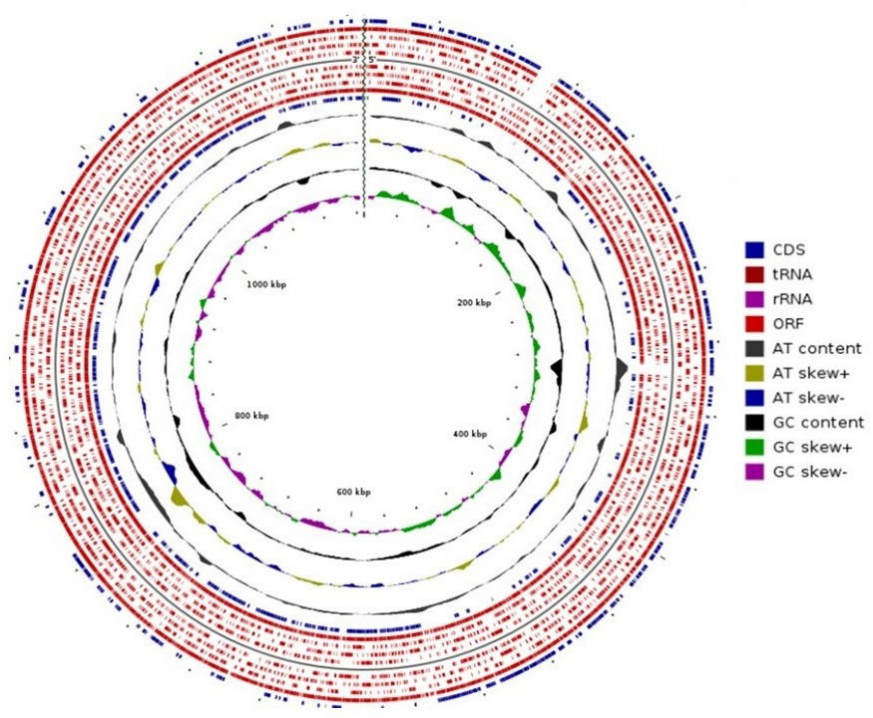
Various regions of the genome of *Bifidobacterium angulatum,* such as confirmed coding region (CDS) annotation, tRNA, rRNA, possible coding region (ORFs) prediction, AT contents, and GC contents. The outermost circle represents the CDS, and inner circles show AT and GC contents.

*B. angulatum* not only acted as a helper strain, promoting the growth of *L. garvieae* and *R. torques*, but it also exhibited growth enhancement when co-cultured with several other strains, including *B. thetaiotaomicron*, *B. longum* subsp*. longum*, and *Bifidobacterium pseudocatenulatum*. Interestingly, while its growth was antagonized by *L. garvieae*, the latter’s growth was, in turn, enhanced by *B. angulatum*, suggesting a dynamic shift in the microbial interactions at play. These intriguing reciprocal relationships prompted us to have a look into the genomic profile of *B. angulatum* through WGS. The main aim of the WGS was to look for potential genetic elements that might be responsible for the observed co- and cross-interactions, as well as the resistome. The WGS analysis revealed that the genome size of *B. angulatum* was 2.09 Mbp having guanine to cytosine ratio (G + C) of 59.64 (Fig. 8).

Genome-wide analysis revealed that this organism carries genes encoding various enzymes that are important for the biosynthesis of various metabolites and may potentially be involved in the observed interactions. Some of the representative examples include the presence of genes encoding acetate kinase and pyruvate formate-lyase, which are involved in the synthesis of acetate and formate and are known to influence microbial behavior. Additionally, the presence of genes encoding adenylosuccinate lyase and argininosuccinate lyase, which are indirectly involved in the production of succinate, is further reported to be involved in complex microbial interactions, such as aiding in the growth of dormant bacteria. Moreover, genes encoding another enzyme, fumarylacetoacetate hydrolase, were present in the genome. This enzyme has a direct role in acetoacetate production, which is considered a key metabolite playing a role in microbial symbiosis as well as competition between members of the bacterial community.

Other genes, the products of which can potentially be involved in the observed interactions, include the biosynthesis of glutamate, transcription repressor ArgR, phosphoserine aminotransferase, and phosphoserine phosphatase-producing genes, which seem to have important roles as per previous reports in cooperative interactions among microbes.

**Fig 9:**
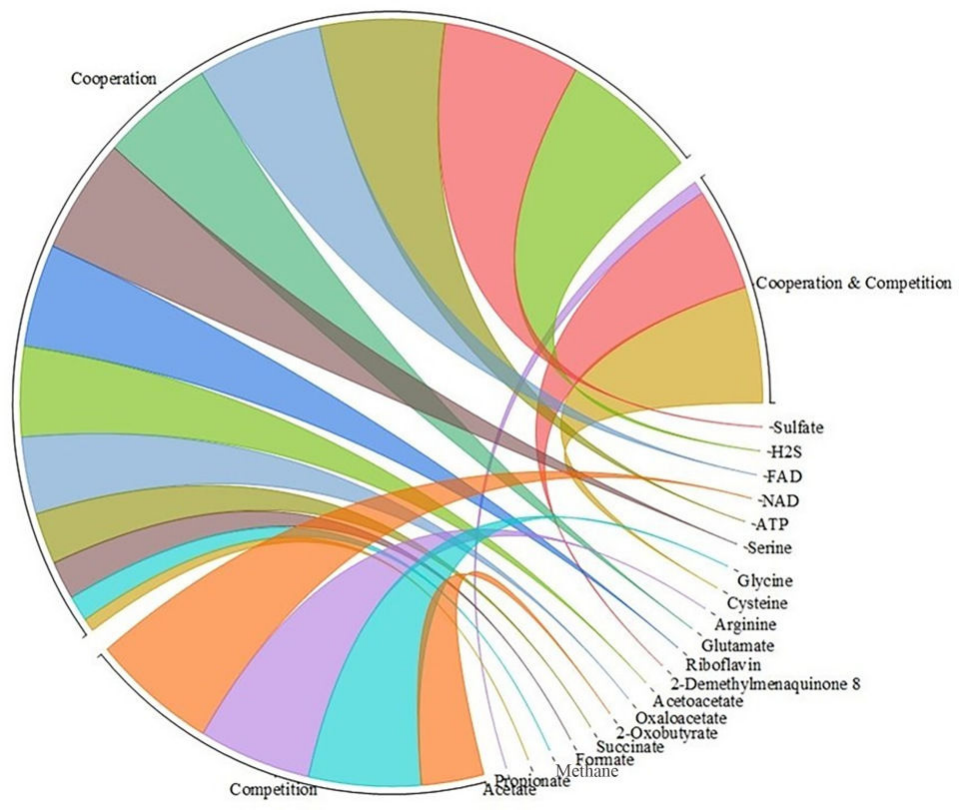
Various metabolites potentially involved in microbe-microbe interactions. The metabolites, as per their functions, are classified into three categories: cooperation, competition, and cooperation/competition.

The metabolic model, constructed using the RAST-annotated genome, highlighted 529 genes and 783 compounds, with 20 metabolites identified as potentially involved in inter-strain interactions. Notably, 65% of these metabolites were associated with cooperative interactions, while 20% exhibited competitive behavior. A remarkable 15% of the metabolites were implicated in both cooperative and competitive roles, mirroring the reciprocal nature of *B. angulatum*’s interactions observed in the phenotypic assays (Fig. 9). This genomic analysis not only provides insights into the metabolic underpinnings of these interactions but also strengthens our understanding of *B. angulatum*’s versatile role within the microbial ecosystem.

## DISCUSSION

The primary objective of this study was to develop a targeted culturomics strategy for isolating core anaerobic bacteria from the healthy human gut and investigate their resistome and interspecies interaction potential. To this end, we successfully optimized a CASGM, formulated to mimic the gut’s unique microenvironment, including its low redox potential, oxygen limitation, and specialized nutrient requirements. Using CASGM, we successfully cultured 21 unique anaerobic bacterial species from the gut, and 11 of these were the core anaerobes. Thus, our CASGM approach successfully targeted the culturing of more than 50% of the core anaerobic species, making it a valuable approach for overcoming the limitations of previous anaerobe-specific culturing approaches (10).

Previously used traditional culturomics approaches, which usually relied on generic anaerobic media types or single batch culture systems, often resulted in limited success as well as did not capture major fractions of the core anaerobes (60). Our CASGM-based approach, on the other hand, utilized a modified media capable of providing stringent anaerobic conditions, which enabled the growth and targeted culturing of a major fraction of the anaerobic organisms. In addition, this strategy resulted in the culturing of previously uncultured taxa from the human gut, thus expanding our knowledge and bioresource of the gut microbiome. To our knowledge, this is the first study that targeted and successfully cultured a major fraction of the core anaerobes by utilizing a single modified growth medium. Thus, our CASGM approach addresses the limitations of the utilization of labor-intensive and costly multi-media approaches and offers a sustainable alternative for targeted culturing of the core anaerobes.

Our comprehensive AMR profiling of the targeted core anaerobes revealed that the majority (55.95%) were susceptible to the tested panel of eight widely used, clinically relevant antibiotics. These include oxytetracycline, meropenem, ampicillin, chloramphenicol, ceftriaxone, levofloxacin, azithromycin, and tobramycin. This is a very valuable finding, suggesting that the majority of the cultured anaerobic strains, both including core and non-core, are vulnerable to the standard antibiotic prescriptions. However, all core anaerobes also revealed resistance to at least two of the tested antibiotics, suggesting that these organisms harbor intrinsic and/or acquired mechanisms that allow them to withstand these antibiotics upon exposure (61).

An interesting observation was that all core isolates were resistant to tobramycin, an aminoglycoside antibiotic. This resistance likely reflects a known pharmacodynamic limitation, as aminoglycosides require oxygen-dependent transport systems for cellular uptake—rendering them ineffective under strict anaerobic conditions such as those present in anaerobic jars (62–66). This AMR snapshot is instrumental in guiding informed antibiotic prescriptions and also reinforces the need for careful selection of antimicrobials in clinical settings to preserve beneficial core taxa and avoid unnecessary microbiome disruption.

Comprehensive cross-interaction profiling among the cultured gut anaerobes revealed an intricate network of ecological behaviors, including synergistic, antagonistic, and bifunctional interactions. The majority of the isolates exhibited measurable effects on growth, highlighting the prevalence of inter-strain influence within the gut ecosystem. Interestingly, 33.33% anaerobic isolates revealed a bifunctional role as they promoted the growth of some taxa but inhibited the growth of others. This further suggests that the cross-interaction behavior of a microbe is not fixed but is context dependent, such as the nutrient availability and the nature of cross-feeding (67, 68). Moreover, various strains showed the role as key facilitators or helper species, each promoting the growth of three or more other strains and termed as major facilitators. These major facilitators include *B. pseudocatenulatum*, *R. inulinivorans*, *M. multacida*, and *B. adolescentis*. One of the strains, *B. pseudocatenulatum,* showed interesting behavior that supported the growth of beneficial strains, including *B. longum* and *L. fujiensis*, while it inhibited the growth of the potentially gut pathogenic strain *P. johnsonii* (3). The potentially pathogenic *L. garvieae* showed antagonistic activities, thereby inhibiting the growth of both various potentially beneficial and harmful strains, suggesting its competitive niche occupant nature (32).

Antagonism (competition) and synergism (cooperation) among the gut microbes *in vivo* within the human gut microbiome have been predicted using modeling, metagenomics, and ecological studies, although not directly observed (8, 69, 70). One of the notable cross-interactions observed was the mutually synergistic relationship between *B. thetaiotaomicron* and *B. adolescentis*. This positive interaction held steady even when the experimental setup was flipped—regardless of which strain was plated first or added to the central well—pointing to a stable mutualism, possibly driven by cross-feeding or shared environmental shaping. Such bidirectional interactions echo previous findings where *B. thetaiotaomicron* modulates gut community structure by liberating polysaccharides and other metabolites utilized by neighboring microbes (71). Interestingly, although *B. thetaiotaomicron* is sometimes associated with disease states, it enhanced the growth of several probiotic taxa, including *B. longum*, *B. angulatum*, *B. adolescentis*, and *Collinsella aerofaciens*, highlighting the limitations of taxonomic classification in inferring functional roles (72).

In a self-contradictory way, various potentially beneficial anaerobes, including *B. pseudocatenulatum*, *M. multacida*, and *R. inulinivorans,* supported the growth of potentially pathogenic bacteria, including *L. garvieae* and *S. anginosus*. This observation further cautions against the traditional binary labeling of strains as probiotic or pathogenic and further suggests that the ecological behavior of a strain in a community strongly depends on the community context (25). Furthermore, *L. formosensis* exhibited both cooperative and antagonistic behaviors—promoting *B. longum* subsp. *longum* while inhibiting six other strains, including *B. coprocola*, *R. torques*, and *B. adolescentis*—demonstrating its dualistic ecological nature.

In summary, our results suggest that a variety of complex interactions exist among the core gut bacteria and within the community, which, in most instances, are position sensitive, and these interactions are governed more by metabolic function and environmental compatibility rather than taxonomy. These findings further support a shift in microbiome-related research from assessment based solely on community structure to microbial interaction-driven frameworks, which can better explain overall community resilience, dysbiosis, and community assembly (67, 72, 73).

Our WGS analysis of the *B. angulatum* strain gives useful insights regarding the potentially responsible genetic element for the observed phenotype. Since this strain displayed a dual role, where, on one hand, it promoted the growth of *L. garvieae* and *R. torques*, on the other hand, some other strains, such as *B. thetaiotaomicron* and *B. longum,* enhanced its growth. Furthermore, WGS analysis revealed a variety of genes and pathways that could potentially be involved in this observed dual behavior. For instance, the presence of genes encoding enzymes that are involved in the synthesis of SCFA (acetate via acetate kinase and formate via pyruvate formate-lyase). Other genes involved in the production of carboxylic acids, such as succinate, oxaloacetate, and several amino acids such as glutamate, serine, arginine, cysteine, and glycine. The above-mentioned metabolic products are well known to be involved in microbial cross-feeding and facilitate and/or affect cooperative behaviors among gut microbial members (14, 74).

Some of the observed synergistic interactions may potentially arise from the secretion of readily available carbon and nitrogen sources by helper bacteria that may be utilized, upon which the neighboring bacteria can thrive. Furthermore, WGS analysis also revealed the presence of genetic elements that might be responsible for the observed competitive behavior. For instance, the presence of genes involved in sulfur metabolism and NAD biosynthesis gives a hint about possible mechanisms through which this strain can monopolize nutrient resources under constrained environments, potentially leading to the inhibition of certain strains. Additionally, the presence of regulatory elements such as ArgR transcription factor (linked to arginine metabolism), along with additional important enzymes such as phosphoserine aminotransferase and phosphoserine phosphatase, indicates the capacity and advantage of this strain for fine-tuned responses based on environmental cues.

Together, these findings suggest that the ecological role of a strain in a community could potentially be predicted from a microbe’s genome (67, 72). *B. angulatum* in our study acts as an interesting example of how genomic elasticity of an organism gives it a multifaceted potential to thrive and survive in the gut ecosystem. This further reinforces the value of the integration of genome-derived data coupled with experimental phenotyping to unleash the functional characteristics of the individual microbes within communities.

### Conclusion

This study presents a unique CASGM strategy for the targeted isolation and characterization of key interaction dynamics as well as the resistome of core anaerobic bacteria of the healthy human gut. By utilizing this targeted single, optimized CASGM approach, we were able to culture a major proportion of the core anaerobic gut species, where some of those were cultured for the first time. Through the combinatorial approach of cross-interaction, AMR profiling, and WGS-resolved functional insights, we uncovered interesting insights into interdependencies and competition among these core strains as well as their ecological flexibilities. Moreover, we observed that some strains, such as *B. thetaiotaomicron* and *B. angulatum,* are capable of either inhibiting or promoting the growth of certain strains depending on the pairing. Such findings go against the traditional binary view of microbes as being purely commensal or pathogenic and suggest that microbial behavior is highly context-dependent. Furthermore, WGS analysis provided useful insights into the potential genomic basis of the observed phenotype. Taken together, our findings provide valuable data and enhance our current understanding of how core anaerobes interact with each other, survive, and adapt within the gut environment. Moreover, our work also lays the critical basis for a more rational design of synthetic microbial communities as well as precision-based microbiome aimed at targeted gut health rehabilitation, both with accuracy and sensitivity.

## Data Availability

The sequences data (V3–V4 region) of this study are available under the accession numbers PX500758–PX500778 in the GenBank, while the whole genome project has been deposited at DDBJ/ENA/GenBank under the accession number JBSGKA000000000.

## References

[1] Sender R, Fuchs S, Milo R. 2016. Revised estimates for the number of human and bacteria cells in the body. PLoS Biol 14:e1002533. doi:10.1371/journal.pbio.1002533 27541692 PMC4991899

[2] Eckburg PB, Bik EM, Bernstein CN, Purdom E, Dethlefsen L, Sargent M, Gill SR, Nelson KE, Relman DA. 2005. Diversity of the human intestinal microbial flora. Science 308:1635–1638. doi:10.1126/science.1110591 15831718 PMC1395357

[3] Lloyd-Price J, Abu-Ali G, Huttenhower C. 2016. The healthy human microbiome. Genome Med 8:51. doi:10.1186/s13073-016-0307-y 27122046 PMC4848870

[4] Maioli TU, Borras-Nogues E, Torres L, Barbosa SC, Martins VD, Langella P, Azevedo VA, Chatel J-M. 2021. Possible benefits of Faecalibacterium prausnitzii for obesity-associated gut disorders. Front Pharmacol 12:740636. doi:10.3389/fphar.2021.740636 34925006 PMC8677946

[5] Tamanai-Shacoori Z, Smida I, Bousarghin L, Loreal O, Meuric V, Fong SB, Bonnaure-Mallet M, Jolivet-Gougeon A. 2017. Roseburia spp.: a marker of health? Future Microbiol 12:157–170. doi:10.2217/fmb-2016-0130 28139139

[6] Hou K, Wu Z-X, Chen X-Y, Wang J-Q, Zhang D, Xiao C, Zhu D, Koya JB, Wei L, Li J, Chen Z-S. 2022. Microbiota in health and diseases. Signal Transduct Target Ther 7:135. doi:10.1038/s41392-022-00974-4 35461318 PMC9034083

[7] Hu C, Shen H. 2024. Microbes in health and disease: human gut microbiota. Appl Sci (Basel) 14:11354. doi:10.3390/app142311354

[8] Armour CR, Nayfach S, Pollard KS, Sharpton TJ. 2019. A metagenomic meta-analysis reveals functional signatures of health and disease in the human gut microbiome. mSystems 4:10. doi:10.1128/mSystems.00332-18 PMC6517693 31098399

[9] Khaledi M, Poureslamfar B, Alsaab HO, Tafaghodi S, Hjazi A, Singh R, Alawadi AH, Alsaalamy A, Qasim QA, Sameni F. 2024. The role of gut microbiota in human metabolism and inflammatory diseases: a focus on elderly individuals. Ann Microbiol 74:1. doi:10.1186/s13213-023-01744-5

[10] Chng KR, Ghosh TS, Tan YH, Nandi T, Lee IR, Ng AHQ, Li C, Ravikrishnan A, Lim KM, Lye D, Barkham T, Raman K, Chen SL, Chai L, Young B, Gan Y-H, Nagarajan N. 2020. Metagenome-wide association analysis identifies microbial determinants of post-antibiotic ecological recovery in the gut. Nat Ecol Evol 4:1256–1267. doi:10.1038/s41559-020-1236-0 32632261

[11] Sun W, Zhang Y, Guo R, Sha S, Chen C, Ullah H, Zhang Y, Ma J, You W, Meng J, Lv Q, Cheng L, Fan S, Li R, Mu X, Li S, Yan Q. 2024. A population-scale analysis of 36 gut microbiome studies reveals universal species signatures for common diseases. NPJ Biofilms Microbiomes 10:96. doi:10.1038/s41522-024-00567-9 39349486 PMC11442664

[12] Faith JJ, Guruge JL, Charbonneau M, Subramanian S, Seedorf H, Goodman AL, Clemente JC, Knight R, Heath AC, Leibel RL, Rosenbaum M, Gordon JI. 2013. The long-term stability of the human gut microbiota. Science 341:1237439. doi:10.1126/science.1237439 23828941 PMC3791589

[13] Xiao Y, Angulo MT, Lao S, Weiss ST, Liu Y-Y. 2020. An ecological framework to understand the efficacy of fecal microbiota transplantation. Nat Commun 11:3329. doi:10.1038/s41467-020-17180-x 32620839 PMC7334230

[14] Louis P, Flint HJ. 2017. Formation of propionate and butyrate by the human colonic microbiota. Environ Microbiol 19:29–41. doi:10.1111/1462-2920.13589 27928878

[15] Rinninella E, Raoul P, Cintoni M, Franceschi F, Miggiano GAD, Gasbarrini A, Mele MC. 2019. What is the healthy gut microbiota composition? a changing ecosystem across age, environment, diet, and diseases. Microorganisms 7:14. doi:10.3390/microorganisms7010014 30634578 PMC6351938

[16] Sommer F, Bäckhed F. 2013. The gut microbiota—masters of host development and physiology. Nat Rev Microbiol 11:227–238. doi:10.1038/nrmicro2974 23435359

[17] Wade WG. 2013. The oral microbiome in health and disease. Pharmacol Res 69:137–143. doi:10.1016/j.phrs.2012.11.006 23201354

[18] Alain K, Querellou J. 2009. Cultivating the uncultured: limits, advances and future challenges. Extremophiles 13:583–594. doi:10.1007/s00792-009-0261-3 19548063

[19] Blaser MJ. 2016. Antibiotic use and its consequences for the normal microbiome. Science 352:544–545. doi:10.1126/science.aad9358 27126037 PMC4939477

[20] Forslund K, Hildebrand F, Nielsen T, Falony G, Le Chatelier E, Sunagawa S, Prifti E, Vieira-Silva S, Gudmundsdottir V, Krogh Pedersen H, et al.. 2015. Disentangling type 2 diabetes and metformin treatment signatures in the human gut microbiota. Nature 528:262–266. doi:10.1038/nature15766 26633628 PMC4681099

[21] Sarsan S, Pandiyan A, Rodhe AV, Jagavati S. 2022. Synergistic interactions among microbial communities, p 1–37. In Singh RP, Manchanda G, Bhattacharjee K, Panosyan H (ed), Microbes in microbial communities: ecological and applied perspectives. Springer.

[22] Hashem ZS. 2025. Bacterial metabolites in defense: a crucial aspect of microbial interaction and host protection, in metabolic dynamics in host-microbe interaction*,* p 101–120. Springer.

[23] Gupta G, Ndiaye A, Filteau M. 2021. Leveraging experimental strategies to capture different dimensions of microbial interactions. Front Microbiol 12:700752. doi:10.3389/fmicb.2021.700752 34646243 PMC8503676

[24] Modi SR, Collins JJ, Relman DA. 2014. Antibiotics and the gut microbiota. J Clin Invest 124:4212–4218. doi:10.1172/JCI72333 25271726 PMC4191029

[25] Buffie CG, Pamer EG. 2013. Microbiota-mediated colonization resistance against intestinal pathogens. Nat Rev Immunol 13:790–801. doi:10.1038/nri3535 24096337 PMC4194195

[26] Almeida A, Mitchell AL, Boland M, Forster SC, Gloor GB, Tarkowska A, Lawley TD, Finn RD. 2019. A new genomic blueprint of the human gut microbiota. Nature 568:499–504. doi:10.1038/s41586-019-0965-1 30745586 PMC6784870

[27] Nayfach S, Roux S, Seshadri R, Udwary D, Varghese N, Schulz F, Wu D, Paez-Espino D, Chen I-M, Huntemann M, et al.. 2021. A genomic catalog of Earth’s microbiomes. Nat Biotechnol 39:499–509. doi:10.1038/s41587-020-0718-6 33169036 PMC8041624

[28] Breznak JA, Costilow RN. 2007. Physicochemical factors in growth. Methods for general and molecular microbiology:309–329. doi:10.1128/9781555817497.ch14

[29] Kageyama A, Benno Y, Nakase T. 1999. Phylogenetic and phenotypic evidence for the transfer of Eubacterium aerofaciens to the genus Collinsella as Collinsella aerofaciens gen. nov., comb. nov. Int J Syst Evol Microbiol 49:557–565. doi:10.1099/00207713-49-2-557 10319476

[30] Yaeshima T, Fujisawa T, Mitsuoka T. 1992. Bifidobacterium species expressing phenotypical similarity to Bifidobacterium adolescentis isolated from the feces of human adults. Bifidobacteria Microflora 11:25–32. doi:10.12938/bifidus1982.11.1_25

[31] Diler O, Altun S, Adiloğlu AK, Kubilay A, Işıklı B. 2002. First occurrence of Streptococcosis affecting farmed rainbow trout (Oncorhynchus mykiss) in Turkey. Bulletin of the European Association of Fish Pathologists 22:21–26.

[32] Collins MD, Farrow JAE, Phillips BA, Kandler O. 1983. Streptococcus garvieae sp. nov. and Streptococcus plantarum sp. nov. Microbiology (Reading, Engl) 129:3427–3431. doi:10.1099/00221287-129-11-3427 6663283

[33] Zakharevich NV, Nezametdinova VZ, Averina OV, Chekalina MS, Alekseeva MG, Danilenko VN. 2019. Complete genome sequence of Bifidobacterium angulatum GT102: potential genes and systems of communication with host. Russ J Genet 55:847–864. doi:10.1134/S1022795419070160

[34] Wang B, Kong Q, Cui S, Li X, Gu Z, Zhao J, Zhang H, Chen W, Wang G. 2021. Bifidobacterium adolescentis isolated from different hosts modifies the intestinal microbiota and displays differential metabolic and immunomodulatory properties in mice fed a high-fat diet. Nutrients 13:1017. doi:10.3390/nu13031017 33801119 PMC8004121

[35] Wong CB, Odamaki T, Xiao J. 2019. Beneficial effects of Bifidobacterium longum subsp. longum BB536 on human health: modulation of gut microbiome as the principal action. J Funct Foods 54:506–519. doi:10.1016/j.jff.2019.02.002

[36] Leser TD, Amenuvor JZ, Jensen TK, Lindecrona RH, Boye M, Møller K. 2002. Culture-independent analysis of gut bacteria: the pig gastrointestinal tract microbiota revisited. Appl Environ Microbiol 68:673–690. doi:10.1128/AEM.68.2.673-690.2002 11823207 PMC126712

[37] Cai Y, Yang J, Pang H, Kitahara M. 2011. Lactococcus fujiensis sp. nov., a lactic acid bacterium isolated from vegetable matter. Int J Syst Evol Microbiol 61:1590–1594. doi:10.1099/ijs.0.025130-0 20675438

[38] Chen Y, Otoguro M, Lin Y, Pan S, Ji S, Yu C, Liou M, Chang Y, Wu H, Yanagida F. 2014. Lactococcus formosensis sp. nov., a lactic acid bacterium isolated from yan-tsai-shin (fermented broccoli stems). Int J Syst Evol Microbiol 64:146–151. doi:10.1099/ijs.0.052811-0 24067730

[39] Appleberry H, Garcia-Israel J, Boger L, Banerjee S, Wolfe AJ, Putonti C. 2025. Streptococcus anginosus of the urogenital tract: evidence of the same strain across anatomical sites of the same females. BMC Genomics 26:840. doi:10.1186/s12864-025-11973-4 41013198 PMC12465951

[40] Zhao L, Wang S, Dong J, Shi J, Guan J, Liu D, Liu F, Li B, Huo G. 2021. Identification, characterization, and antioxidant potential of Bifidobacterium longum subsp. longum strains isolated from feces of healthy infants. Front Microbiol 12:756519. doi:10.3389/fmicb.2021.756519 34795651 PMC8593421

[41] Lu W, Pei Z, Zang M, Lee Y-K, Zhao J, Chen W, Wang H, Zhang H. 2021. Comparative genomic analysis of Bifidobacterium bifidum strains isolated from different niches. Genes (Basel) 12:1504. doi:10.3390/genes12101504 34680899 PMC8535415

[42] Ulger Toprak N, Celik C, Cakici O, Soyletir G. 2004. Antimicrobial susceptibilities of Bacteroides fragilis and Bacteroides thetaiotaomicron strains isolated from clinical specimens and human intestinal microbiota. Anaerobe 10:255–259. doi:10.1016/j.anaerobe.2004.05.005 16701525

[43] Hisatomi A, Tourlousse DM, Hamajima M, Ohkuma M, Sekiguchi Y, Sakamoto M. 2023. Complete genome sequences of Ruminococcus torques strains JCM 36208 and JCM 36209, isolated from the feces of a healthy Japanese male. Microbiol Resour Announc 12:e0063223. doi:10.1128/MRA.00632-23 37800929 PMC10652952

[44] Johnson JL, Moore WEC, Moore LVH. 1986. Bacteroides caccae sp. nov., Bacteroides merdae sp. nov., and Bacteroides stercoris sp. nov. isolated from human feces. Int J Syst Bacteriol 36:499–501. doi:10.1099/00207713-36-4-499

[45] Kitahara M, Sakamoto M, Ike M, Sakata S, Benno Y. 2005. Bacteroides plebeius sp. nov. and Bacteroides coprocola sp. nov., isolated from human faeces. Int J Syst Evol Microbiol 55:2143–2147. doi:10.1099/ijs.0.63788-0 16166722

[46] Bakir MA, Kitahara M, Sakamoto M, Matsumoto M, Benno Y. 2006. Bacteroides intestinalis sp. nov., isolated from human faeces. Int J Syst Evol Microbiol 56:151–154. doi:10.1099/ijs.0.63914-0 16403880

[47] Dai W, Zhang J, Chen L, Yu J, Zhang J, Yin H, Shang Q, Yu G. 2023. Discovery of Bacteroides uniformis F18-22 as a safe and novel probiotic bacterium for the treatment of ulcerative colitis from the healthy human colon. Int J Mol Sci 24:14669. doi:10.3390/ijms241914669 37834117 PMC10572632

[48] Gómez Del Pulgar EM, Benítez-Páez A, Sanz Y. 2020. Safety assessment of Bacteroides uniformis CECT 7771, a symbiont of the gut microbiota in infants. Nutrients 12:551. doi:10.3390/nu12020551 32093252 PMC7071458

[49] Sakamoto M, Kitahara M, Benno Y. 2007. Parabacteroides johnsonii sp. nov., isolated from human faeces. Int J Syst Evol Microbiol 57:293–296. doi:10.1099/ijs.0.64588-0 17267966

[50] Duncan SH, Aminov RI, Scott KP, Louis P, Stanton TB, Flint HJ. 2006. Proposal of Roseburia faecis sp. nov., Roseburia hominis sp. nov. and Roseburia inulinivorans sp. nov., based on isolates from human faeces. Int J Syst Evol Microbiol 56:2437–2441. doi:10.1099/ijs.0.64098-0 17012576

[51] Hodge HM, Sherman JM. 1937. Streptococcus equinus. J Bacteriol 33:283–289. doi:10.1128/jb.33.3.283-289.1937 16559995 PMC545391

[52] Vallianou NG, Kounatidis D, Tsilingiris D, Panagopoulos F, Christodoulatos GS, Evangelopoulos A, Karampela I, Dalamaga M. 2023. The role of next-generation probiotics in obesity and obesity-associated disorders: current knowledge and future perspectives. Int J Mol Sci 24:6755. doi:10.3390/ijms24076755 37047729 PMC10095285

[53] Cheng Z, Huang Y, Wie W, Wang Y, Wang Z. 2019. Bloodstream infection caused by bacteroides caccae in a diabetic patient: a case report and review of the literature. Clin Lab 65:12. doi:10.7754/Clin.Lab.2019.190534 31850719

[54] Duranti S, Milani C, Lugli GA, Mancabelli L, Turroni F, Ferrario C, Mangifesta M, Viappiani A, Sánchez B, Margolles A, van Sinderen D, Ventura M. 2016. Evaluation of genetic diversity among strains of the human gut commensal Bifidobacterium adolescentis. Sci Rep 6:23971. doi:10.1038/srep23971 27035119 PMC4817515

[55] Ku S, Haque MA, Jang MJ, Ahn J, Choe D, Jeon JI, Park MS. 2024. The role of Bifidobacterium in longevity and the future of probiotics. Food Sci Biotechnol 33:2097–2110. doi:10.1007/s10068-024-01631-y 39130652 PMC11315853

[56] Underwood MA, German JB, Lebrilla CB, Mills DA. 2015. Bifidobacterium longum subspecies infantis: champion colonizer of the infant gut. Pediatr Res 77:229–235. doi:10.1038/pr.2014.156 25303277 PMC4350908

[57] Gu M, Zhang Z, Pan C, Goulette TR, Zhang R, Hendricks G, McClements DJ, Xiao H. 2019. Encapsulation of Bifidobacterium pseudocatenulatum G7 in gastroprotective microgels: improvement of the bacterial viability under simulated gastrointestinal conditions. Food Hydrocoll 91:283–289. doi:10.1016/j.foodhyd.2019.01.040

[58] Soltani M, Jamshidi S, Sharifpour I. 2005. Streptococcosis caused by Streptococcus iniae in farmed rainbow trout (Oncorhynchys mykiss) in Iran: biophysical characteristics and pathogenesis. Bulletin of the European Association of Fish Pathologists 25:95–106.

[59] Liu J, Zhang Y, Xu L, Gu G, Dong Z. 2025. Parabacteroides johnsonii inhibits the onset and progression of colorectal cancer by modulating the gut microbiota. J Transl Med 23:734. doi:10.1186/s12967-025-06675-0 40605035 PMC12225111

[60] Kapinusova G, Lopez Marin MA, Uhlik O. 2023. Reaching unreachables: obstacles and successes of microbial cultivation and their reasons. Front Microbiol 14:1089630. doi:10.3389/fmicb.2023.1089630 36960281 PMC10027941

[61] Hecht DW. 2004. Prevalence of antibiotic resistance in anaerobic bacteria: worrisome developments. Clin Infect Dis 39:92–97. doi:10.1086/421558 15206059

[62] Bryan LE, Kowand SK, Van Den Elzen HM. 1979. Mechanism of aminoglycoside antibiotic resistance in anaerobic bacteria: Clostridium perfringens and Bacteroides fragilis. Antimicrob Agents Chemother 15:7–13. doi:10.1128/AAC.15.1.7 218500 PMC352592

[63] Ramirez MS, Tolmasky ME. 2010. Aminoglycoside modifying enzymes. Drug Resist Updat 13:151–171. doi:10.1016/j.drup.2010.08.003 20833577 PMC2992599

[64] Schlessinger D. 1988. Failure of aminoglycoside antibiotics to kill anaerobic, low-pH, and resistant cultures. Clin Microbiol Rev 1:54–59. doi:10.1128/CMR.1.1.54 3060245 PMC358029

[65] Martin WJ, Gardner M, Washington JA II. 1972. In vitro antimicrobial susceptibility of anaerobic bacteria isolated from clinical specimens. Antimicrob Agents Chemother 1:148–158. doi:10.1128/AAC.1.2.148 4680804 PMC444184

[66] Kislak JW. 1972. The susceptibility of Bacteroides fragilis to 24 antibiotics. J Infect Dis 125:295–299. doi:10.1093/infdis/125.3.295 4335792

[67] Venturelli OS, Carr AV, Fisher G, Hsu RH, Lau R, Bowen BP, Hromada S, Northen T, Arkin AP. 2018. Deciphering microbial interactions in synthetic human gut microbiome communities. Mol Syst Biol 14:e8157. doi:10.15252/msb.20178157 29930200 PMC6011841

[68] Friedman J, Higgins LM, Gore J. 2017. Community structure follows simple assembly rules in microbial microcosms. Nat Ecol Evol 1:109. doi:10.1038/s41559-017-0109 28812687

[69] Jansma J, El Aidy S. 2021. Understanding the host-microbe interactions using metabolic modeling. Microbiome 9:16. doi:10.1186/s40168-020-00955-1 33472685 PMC7819158

[70] Luo M, Zhu J, Jia J, Zhang H, Zhao J. 2024. Progress on network modeling and analysis of gut microecology: a review. Appl Environ Microbiol 90:e00092–24. doi:10.1128/aem.00092-24 38415584 PMC11207142

[71] Rakoff-Nahoum S, Foster KR, Comstock LE. 2016. The evolution of cooperation within the gut microbiota. Nature 533:255–259. doi:10.1038/nature17626 27111508 PMC4978124

[72] Fernandez-Julia P, Commane DM, van Sinderen D, Munoz-Munoz J. 2022. Cross-feeding interactions between human gut commensals belonging to the Bacteroides and Bifidobacterium genera when grown on dietary glycans. Microbiome Res Rep 1:12. doi:10.20517/mrr.2021.05 38045648 PMC10688802

[73] Coyte KZ, Schluter J, Foster KR. 2015. The ecology of the microbiome: networks, competition, and stability. Science 350:663–666. doi:10.1126/science.aad2602 26542567

[74] Magnúsdóttir S, Heinken A, Kutt L, Ravcheev DA, Bauer E, Noronha A, Greenhalgh K, Jäger C, Baginska J, Wilmes P, Fleming RMT, Thiele I. 2017. Generation of genome-scale metabolic reconstructions for 773 members of the human gut microbiota. Nat Biotechnol 35:81–89. doi:10.1038/nbt.3703 27893703

